# Unlike Twins: An NMR Comparison of Two α-Synuclein Polymorphs Featuring Different Toxicity

**DOI:** 10.1371/journal.pone.0090659

**Published:** 2014-03-05

**Authors:** Julia Gath, Luc Bousset, Birgit Habenstein, Ronald Melki, Anja Böckmann, Beat H. Meier

**Affiliations:** 1 Physical Chemistry, ETH Zürich, Zurich, Switzerland; 2 Laboratoire d′Enzymologie et Biochimie Structurales, UPR 3082 CNRS, Gif-sur-Yvette, France; 3 Institut de Biologie et Chimie des Protéines, UMR 5086 CNRS/Université de Lyon 1, Lyon, France; University of Pittsburgh School of Medicine, United States of America

## Abstract

We structurally compare, using solid-state NMR, two different polymorphs of α-synuclein which, as established recently, display contrasting biochemical properties, toxicity, and tropism for cells. We show that both forms, which can each be produced as a pure polymorph, are greatly different in secondary structure. While β-sheets are the dominating secondary structure elements for both polymorphs, they are markedly divergent in terms of number of elements, as well as their distribution. We demonstrate that all identified β-sheets feature an in-register parallel stacking for both polymorphs. The two forms show a different molecular arrangement in the unit cell and distinct dynamic features, while sharing a highly flexible C-terminal domain. The use of reproducible, well-identified conditions for sample preparation and the recording of identical NMR experiments allows for a direct comparison of the results.

## Introduction

Fibrils are large protein assemblies that are deposited intra- or extracellularly in a number of human diseases termed proteinopathies, which include Parkinson's, Alzheimer's, Huntington's, and Creutzfeldt-Jacob diseases. [1], [2] In Parkinson's disease, the second-most frequent neurodegenerative disease in humans, the protein aggregated predominantly into so-called Lewy bodies and Lewy neurites [3] is α-synuclein, a 140 amino-acid residue protein which shall be investigated in the following.

It has been realized over the last years that polymorphism is present for many fibrillar proteins (e.g. in Aβ[4], [5]) and also for α-synuclein. [6], [7] The existence of polymorphs is an interesting phenomenon which has been linked to the phenomenon of strains in prions[8]. The fact that α-synuclein spreads within an organism by cell-to-cell transmission, and the existence of different synucleinopathies [9], [10] may possibly be linked to a prion-like behavior of the different polymorphs. [11], [12]

Polymorphism is manifested by differences in fibril morphology seen in electron micrographs, qualitatively different x-ray fiber diffraction patterns, and by solid-state NMR chemical shifts which are highly sensitive to conformational differences. In NMR, polymorphism can be manifest by line broadening typical for small structural heterogeneities, or by traceable chemical-shift differences of up to several ppm for structurally distinct forms as well as different intensities in certain spectra. Different polymorphs can have different biophysical properties and cellular responses and may be the structural bases of different strains in amyloids and prions. [12] In the absence of a detailed quality control, samples may exist in a mixture of several polymorphs. In this context, solid-state NMR can be an indispensible analytical tool, but can, even more importantly, yield detailed structural and dynamic information.

While there may be cases where extensive polymorphism seems unavoidable, like the isolated prion domain of Ure2p[13]–[15], we show in the following that two structurally different polymorphs of α-synuclein can be obtained in pure form allowing a thorough characterization by NMR. Importantly, these polymorphs also display different toxicity, as well as different *in vivo* and *in vitro* seeding and propagation properties, and may delineate different types of synucleinopathies.[12] While results obtained on different α-synuclein polymorphs in different labs have been compared before,[16] we concentrate here on characterizing two samples of human α-synuclein, where everything, from the expression protocol to the NMR experimental parameters was identical, except the salt content of the fibrillization buffer, and whose biochemical and biophysical differences have been characterized and assigned to two different strains. Indeed, it has been observed over the last years that sample preparation protocols, as well as the use of different NMR experiments and equipment yielding different signal-to-noise characteristics and different sensitivity to dynamic effects has lead to a panoply of data which are disconcerting to compare. This is particularly true for the N-terminal part of synuclein, which in the two forms we describe here is found to be fully and partially ordered respectively, and thus shows important differences to the previously characterized polymorphs. Recent results indicate that the N-terminal part, which behaves entirely different in the two polymorphs, plays an important role in the seeding behavior of synuclein in a mouse model. [11]

## Results

### Two pure polymorphs

**Figure 1:**
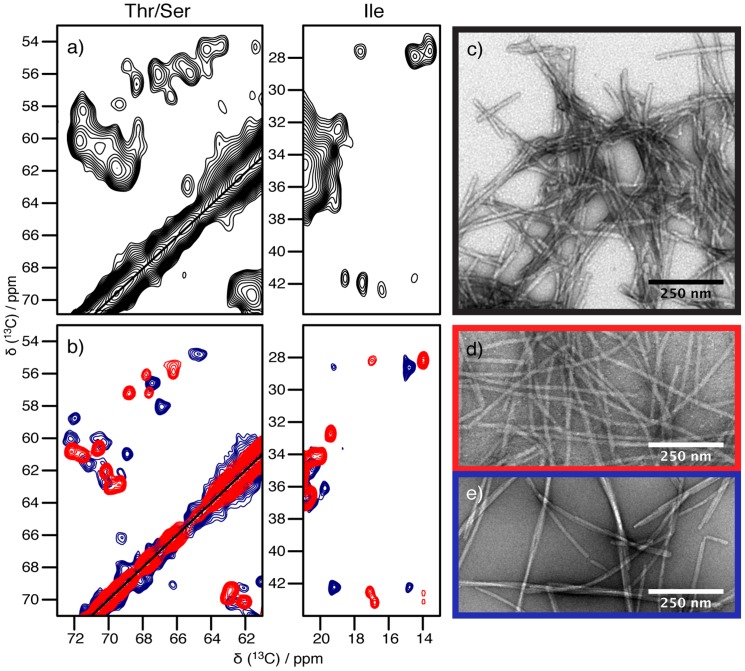
Extracts of 20 ms DARR spectra highlight the sample quality. All NMR spectra are from uniformly [^13^C,^15^N]-labeled α-synuclein fibrils and the electron micrographs are taken from the same batch of sample. (a) An early sample preparation resulting in a mixture of polymorphs. The spectrum was recorded at 14.1 T static magnetic field and 13 kHz MAS. (b) Spectra of preparations of pure polymorphs. Ribbons are shown in blue and fibrils in red. The spectra were recorded at 20.0 T static magnetic field and 17 kHz MAS. Full spectra are provided in ref. [20], [21]. All resonances of the fibrils and the ribbons plus additional resonances are present in the spectrum of the mixture.

**Figure 2:**
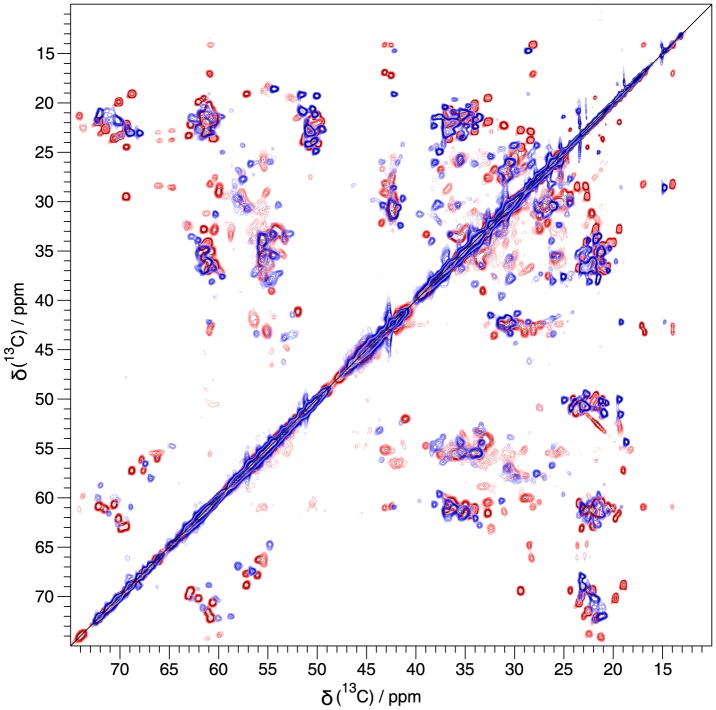
Ribbon and fibril feature entirely different spectra. Overlay of the aliphatic region of a 20 ms DARR spectra of U [^13^C,^15^N] labeled α-synuclein ribbons (blue)[20] and α-synuclein fibrils (red)[21]. The spectra were recorded at 20.0 T static magnetic field and 17 kHz MAS and processed with a shifted sine-bell window function (SSB 2.6). The fibril spectrum is based on the same time-domain data as the DARR used for assignment. Individual spectra (with a slightly different processing) are given in the assignment notes [20], [21].

Figure 1 compares extracts from 2D ^13^C correlation spectra of different preparations of α-synuclein fibrils. While Figure 1a displays in black spectral extracts of an early sample, Figure 1b presents an overlay of two more recent samples, containing the pure polymorphs employed in this study. Comparing the three spectra, one easily realizes that the spectrum of the early sample (black contours) presents a mixture of the two polymorphs, called ribbons and fibrils according to ref [12] and given in blue and red contours, respectively, and possibly further polymorphs. The spectrum of the mixture shown in Figure 1a is quite impossible to analyze in detail, as most resonances overlap, even though the individual resonances buried in the broad spectral features are probably as narrow as in the preparations highlighted in blue and red. Counting of spin systems present in the spectrum clearly results in excess resonances with respect to the expected number of particular amino-acid residues, as for example there are signals from three isoleucine spin systems observed, with only two isoleucine residues present in the primary sequence. Furthermore, there are four serine residues, but at least eight cross peaks are observed in the serine Cα-Cβ region. The spectra of the pure polymorphs, in contrast, show mostly isolated resonances with narrow linewidth, rivaling spectra of microcrystalline preparations.[17]–[19] EM pictures of the respective fibrils are given in Figure 1c,d and e. The full aliphatic regions of the DARR spectra for ribbons and fibrils are found in the assignment notes [20], [21] and the one of the mixture in Figure S1 (mixture). An overlay of the spectra of the two pure polymorphs is shown in Figure 2. As for their different appearance in electron micrographs we refer to the red form as “fibrils”, and to the blue form as ribbons, due to their flat and twisted morphology.[12] Throughout the results discussed in the following, data referring to fibrils are depicted in red, and those obtained from ribbons in blue.

Two different buffer conditions reproducibly produce fibrils and ribbons: aggregation under physiological salt conditions (150 mM KCl) yield fibrils, while ribbons are obtained under low salt conditions. Both polymorphs fibrillize at neutral pH. Other preparations of pure polymorphs of human α-synuclein have been described in [22] (possibly identical to the ribbon form) and in[23]–[26]. In the latter case, nominally identical preparations are used; however the NMR spectral fingerprint of the fibrils obtained is different in reference [24] from the ones shown in refs [25], [26], pointing to different polymorphs.

### Secondary structures of fibrils and ribbons differ greatly

**Figure 3:**
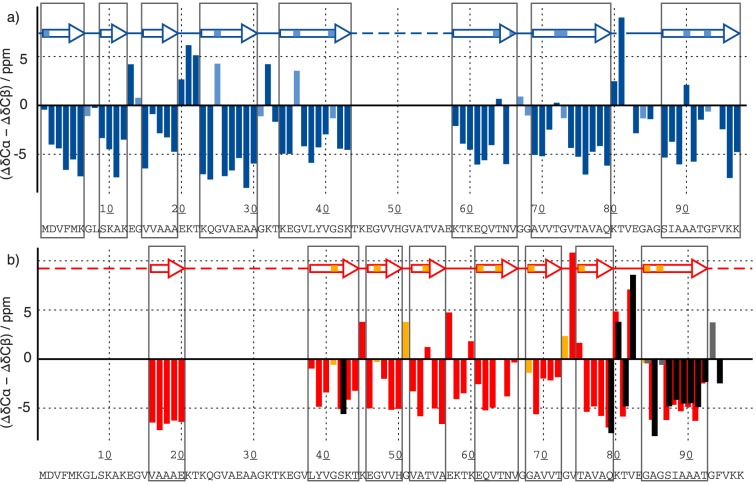
Secondary structure analysis. Differences between secondary chemical shifts of Cα and Cβ resonances relative to their random-coil shifts as tabulated by Wishart and Sykes [27] of the sequentially assigned residues of α-synuclein. The data from the BMRB entries 17498 (ribbons) and 18860 (fibrils) were used for the plot. The secondary chemical shifts are shown in (a) blue for the ribbons and in (b) red and black for the fibrils, where black stands for the second of the doubled resonances starting at residue 79. The individual plots for the two polymorphs have already be presented in ref. [20], [21] and are replotted here to facilitate a direct comparison. The data for the ribbons differ slightly do to improved data obtained at 20 T. For glycine residues, only the deviation of the Cα shift from the random coil value is displayed. Glycines are indicated in light blue (ribbons) and in orange and grey (fibrils) for chain A and chain B, respectively. β-strands, as defined by three or more non-glycine residues in a row with a negative difference of the secondary shifts between Cα and Cβ are marked with arrows in dark blue or red, and lighter colors are used where glycine residues, which possibly are included into β-sheet, or where TALOS [63] predicted β-sheet, despite that the secondary chemical shift difference was positive. We only consider the dark stretches as trusted β sheets.

Carbon-13 backbone chemical shifts in proteins are very sensitive to the backbone dihedral angles φ and Ψ, allowing for the identification of α-helical and β-sheet secondary structure elements. The difference between the deviations of the Cα and the Cβ shifts from their random-coil value[27] are a proxy for secondary structure: Four or more positive values in a row indicate α-helical secondary structure, while at least three negative values in a row indicate β-sheets. The secondary chemical-shift analysis based on the assignments deposited in the BMRB (17498 (ribbons) and 18860 (fibrils)) for the two polymorphs is shown in Figure 3. The secondary structure plot reveals a high β-strand content, a hallmark of amyloid fibrils, for both polymorphs. β-strands are marked with arrows in the figure. Dark red or blue parts of the arrows refer to sheets following the above definition where Gly residues were not considered. In the HET-s amyloid, where an atomic resolution structure is available [28], [29], glycine residues were always found in turns, even if the Cα secondary shift indicated a β-strand conformation. [30] Furthermore glycine residues in other amyloids often lead to warnings when TALOS predictions are applied (unpublished data). Therefore we treat all glycines as possibly marking turns and mark possible extensions of the sheets defined that way either by Gly residues or other residues with a positive shift difference but a β sheet prediction by TALOS with lighter color (Figure 3). Details of the assignments are described in the assignment notes. [20], [21]. The extent of assignments, as well as the resonance position of the assigned signals, varies considerably between the two polymorphs, but the observation of intense, narrow signals in all 3D spectra used for assignments means that the two polymorphic forms are well defined and highly ordered. The assigned signals refer to rigid parts of the fibrils. In contrast, missing signals indicate that the corresponding amino-acid residues display dynamic behavior. Important differences can be observed for the two polymorphs. In the case of the fibrils, the core region ranges from Val37 to Phe94, believed to constitute the fibrillar core of α-synuclein.[31], [32] In addition, a number of weaker signals from a β-strand including residues 16–20 are observed and assigned. For the ribbons, however, the entire N-terminal part of the protein, down to residue 1, is rigid and ordered into β-sheets. Also, the patterns of the β-strands vary between the two polymorphs for the region which is rigid and NMR-visible in both polymorphs (residue 58 to 94). The location of arcs and turns is clearly different between the two polymorphs. In general, the ribbons seem to have a tendency to form long β-strands, while the β-strand pattern in the fibrils is less regular, with many shorter β-strands. Interestingly, the N-terminal pseudo-repeat units are not reflected in the secondary chemical shifts of either polymorph, indicating that they do not lead to repeating structural features.

### Ribbons have one molecule per asymmetric unit—fibrils have two

**Figure 4:**
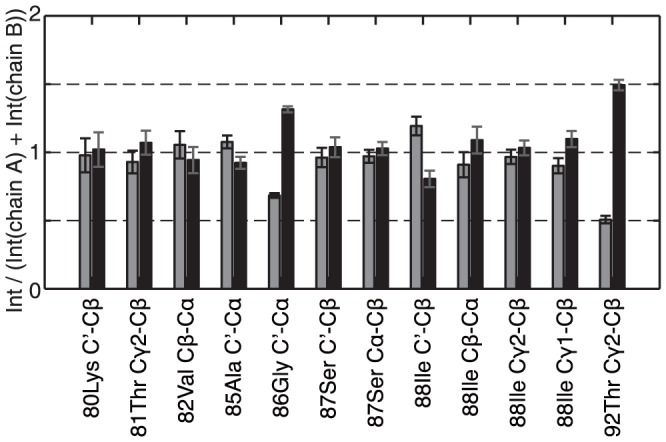
Chains A and B signals have the same intensity. Intensity ratio for selected doubled peaks using a 20 ms DARR spectra. Chain A is shown in black and chain B in grey. The errors are estimated from the experimental noise. All residues show a ratio close to 1:1 except for Gly86 and Tyr92 where the two forms may feature slightly different mobility.

For fibrils, as for crystals, we define an asymmetric unit which contains a minimum set of atoms from which, by symmetry, the fibril can be constructed. For synuclein, the asymmetric unit contains one or more molecules. For ribbons, only a single set of resonances is observed, indicating that there is only one molecule in the asymmetric unit. Then, all molecules are chemically equivalent and have the same resonance frequencies in a MAS NMR experiment. In the fibrils, peak doubling is observed between residues 79 to 94, towards the end of the core region (see Figure 3), pointing at two different conformations. In addition, Cβ of Ser42 is doubled. Those peaks were assigned to two different chains, A and B. In general, the NH and Cβ shifts are affected most by the peak doubling. However, the secondary chemical shifts remain very similar, indicating that the secondary structure of those two chains is similar, with only limited changes of the dihedral angles. Actually, alternatively to pointing to two slightly different molecules in the asymmetric unit, peak doubling could also indicate the presence of locally restricted polymorphism, e.g the presence of two polymorphic fibrils with a single conformation each. The observation however, illustrated in Figure 4, that the cross peak ratios of the two forms are all close to 1, and show the same intensity pattern, clearly points to the a asymmetric unit with two molecules. We analyzed this ratio for different batches of fibril samples, and no significant batch dependence could be observed as would be expected for a mixture of polymorphs. The cross peak ratios for four further batches of α-synuclein fibrils are shown in Figure S2.

### Fibrils and ribbons both form in-register parallel β-sheets

**Figure 5:**
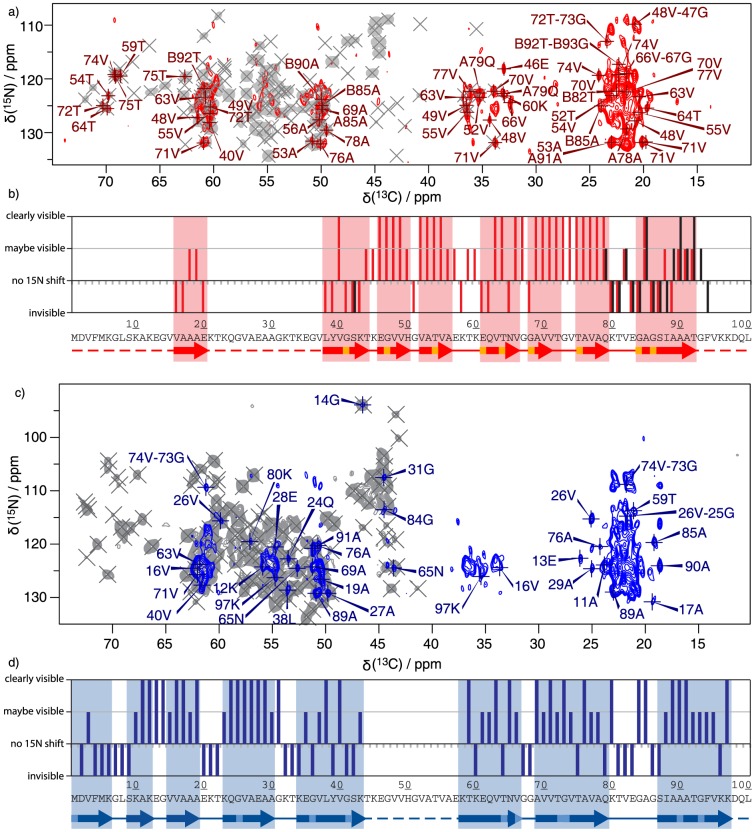
Ribbons and fibrils have an in-register parallel stacking. (a) and (c) NCA (grey) and PAIN (red/blue) spectra of the fibril and the ribbon form, respectively. Peak maxima of the NCA spectra, taken from uniformly labeled samples, are labeled with grey crosses. Peaks that could unambiguously be assigned are labeled in the PAIN spectra obtained from 1:1 mixture of ^13^C and ^15^N labeled monomers. All spectra were recorded at 20.0 T static magnetic field and 17 kHz MAS. (b) and (d). Contacts visible in the PAIN spectrum are plotted against the sequence. ‘Clearly visible’ peaks could be assigned unambiguously in the PAIN and are labeled in (a) and (c), for ‘maybe visible’ ones there is intensity in the PAIN, however several assignments are possible, for ‘invisible’ ones no intensity could be observed at the expected positions. Regions supposedly in β-sheets regions according to TALOS and their secondary chemical shifts are highlighted in light red and light blue.

Figure 5a shows a ^13^C-^15^N correlation spectrum of α-synuclein in fibril form obtained from a 1∶1 mixture of ^13^C-labeled and ^15^N-labelled α-synuclein monomers. The corresponding spectrum for the ribbons is given in Figure 5c. The spectra were obtained using the PAIN pulse sequence[33] which leads to ^15^N-^13^C polarization transfer by a cross term containing the ^13^C/^15^N heteronuclear couplings to a nearby proton spin.[33]–[35] The PAIN spectra of the fibril form is, in Fig 5a, overlaid with an NCA correlation spectrum of a uniformly labeled sample of the same polymorphic form. The observed PAIN N-Cα correlations, which must be of intermolecular origin, are the same as observed in the NCA spectrum and assigned to intramolecular next-neighbor contacts. For the β-sheet parts of the structure, these contacts establish the so-called register contacts, which refers to contacts with the hydrogen-bonded residue in the adjacent β-strand. Our results therefore establish an in-register parallel stacking for most of the detected β-sheets in the fibril form (see Figure 5b). That not all expected N-Cα correlations are observed in the PAIN spectrum is due to the different polarization-transfer characteristics of a NCA-CP and a PAIN-CP. In Figure S3 an overlay of the mixed labeled and PAIN with the reference spectrum recorded on a uniformly labeled sample is shown. Here, all transfer parameters are kept the same. Both samples yield virtually the same spectra, showing that the fibrils form of α-synuclein exhibits parallel in register stacking. Figure 5b plots the contacts visible in the mixed labeled PAIN of the fibril form against the sequence. Those contacts are well distributed over the whole rigid core of the sequence. Regions supposedly in β-sheets are more often identified in the PAIN than others. However, this might be due to their higher signal-to-noise ratio.

The PAIN spectrum obtained on a ^13^C/^15^N mixed labeled sample compared to an NCA spectrum of the ribbon form is shown in Figure 5c. A comparison to a PAIN spectrum of uniformly [^13^C,^15^N]-labeled α-synuclein ribbons is given in Figure S4. Also for this sample, in-register parallel contacts are found all along the assigned stretches of the sequence (see Figure 5d). An in-register parallel packing has also be found in[22] and indeed their sample may be very similar to the ribbons form.

### β-sheet core residues show variable rigidity

Signal intensities of NCA or CANCO cross peaks are a rough and qualitative measure for the presence of local dynamics,[24], [36], [37] For α-synuclein, 3D spectra are needed to obtain the necessary resolution and, indeed, amongst the 3D assignment spectra,[38] the CANCO spectrum is best suited for such a study, as there is one cross peak per residue, and the intensity does not depend on the length and type of the side chain. All transfer steps used are CP steps. The intensity is mainly determined by T_1ρ_ relaxation time and therefore, can reports qualitatively on the molecular dynamics of the corresponding residue.

**Figure 6:**
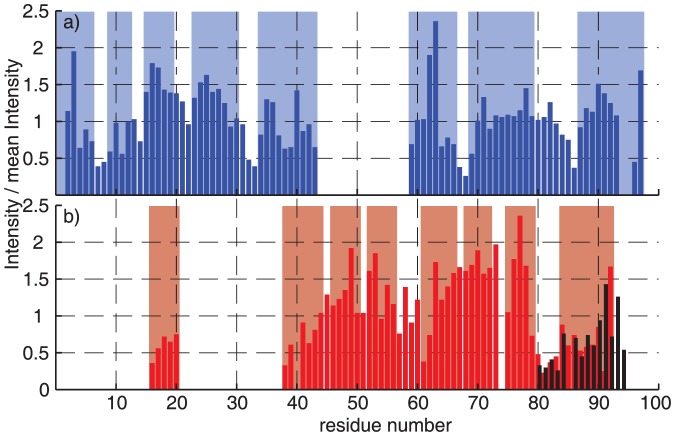
The dynamics correlates with secondary-structure elements. Intensity profile of CANCO cross peaks for (a) ribbons (blue) and (b) fibrils (red). The intensity reflects the local mobility, as all transfers used are CP steps and are therefore the intensity depends on T_1ρ_. Regions that are believed to be in β-sheets are highlighted.

Figure 6 shows a CANCO cross-peak intensity profile for α-synuclein ribbons and fibrils. The pattern of the dynamics globally follows the secondary-structure elements. Signals arising from residues in β-strand secondary structure are in general more intense than the connecting residues. The only exceptions are residues 80 to 85 in the ribbons. Those are as intense as the preceding β-strand, but their secondary chemical shifts only vary little from the random coil values and no clear TALOS prediction could be obtained. Interestingly, in the ribbons, the N-terminal portion is as intense as the so-called NAC region[39] pointing to equal rigidity. On the other hand, in the fibrils, the short N-terminal β-strand (residues 16-20) is seen only half as intense as the core region. Also, the intensity progressively drops within the second β-strand from residue 44 to residue 38. The N-terminus of the fibrils seems to be more mobile than the core. The intensity profile of the fibrils resembles very much the intensity profile published by Comellas *et al.* on another polymorph of α-synuclein.[24] Thus, these two forms do not only have a similar extent of structured core residues, but also are close in their dynamic properties. The ribbons, in contrast, resemble neither of the other polymorphs characterized so far by solid-state NMR.

### The C-terminal residues of fibrils and ribbons are flexible

**Figure 7:**
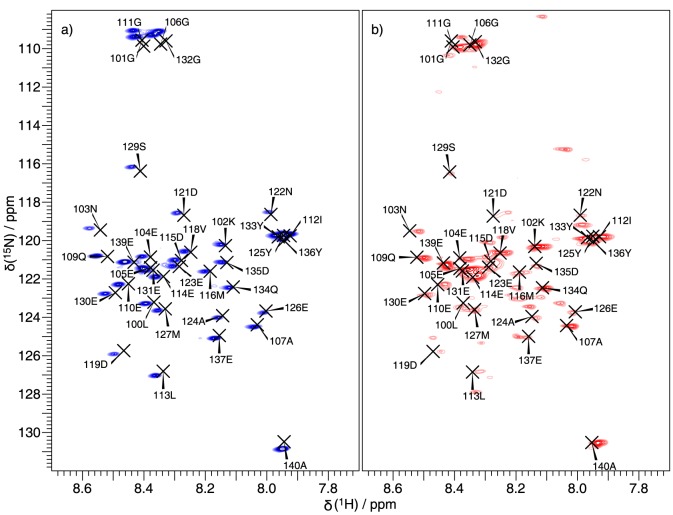
Residues 100 to 140 are highly dynamic. ^1^H-^15^N spectra of (a) ribbons (blue) and (b) fibrils (red). Black crosses mark the assigned resonances of the ribbon sample. The solution-state assignment of reference[42] can essentially be adopted but the assignment was verified with an HNCA assignment spectrum. All residues from Leu100 to the C-terminus, with exception of the prolines, are observed in the spectrum for the ribbons. For fibrils, these resonances are present as well. The black crosses in (b) mark the corresponding peak positions of the C-terminal resonances in the ribbon sample. Additional, weaker, signals are observed for fibrils, which likely come from α-synuclein monomers (see Figure S5 for details).

No cross-polarization-based NMR signals were detected for residues 100 to 140, neither for fibrils nor for ribbons. In contrast, these residues can be detected using “solution-state” HSQC experiments.[40] Still, the application of MAS plays an important role to average susceptibility effects.[41] In the HSQC spectra of the ribbons (Figure 7a), we detect 36 narrow backbone resonances for residues 100 to 140 (all except the prolines) at the virtually identical resonance positions as in the spectrum of dissolved α-synuclein monomers as previously published (BMRB 16543)[42], and with resonance frequencies compatible with a disordered random-coil structure. For the fibrils, the same resonances are also present (Figure 7b), and they lead to the strongest resonances in the HSQC spectrum. In addition, and in contrast with the ribbons, we also observed signals from residues 1–100, at lower intensity, which we explain by the presence of α-synuclein monomers in the sample. These observations agree with our recent biochemical data that show that fibrils disassemble within hours when cooled to 4°C, while ribbons hardly disassemble. [12] An HSQC plotted at lower contour levels, emphasizing these monomer signals. is shown in Figure S5. Some resonances that could be unambiguously assigned based on the proton and nitrogen frequencies and with the solution-state chemical shifts [42] as reference, are labeled in the Figure.

## Materials and Methods

### Sample preparation

Soluble, monomeric a-syn in 20mM Tris, pH 7.5, 350mM KCl was dialyzed against 5mM Tris pH 7.5 for 16h at 4°C. The salt concentration was then adjusted to 150mM KCl prior to assembly. This early assembly procedure yielded samples with different polymorphs present in one batch.

When soluble, monomeric a-syn in 20mM Tris, ph 7.5, 350m MKCl was split into two aliquots, one dialyzed against 20mM Tris, ph 7.5, 150 mM KCl for 16h, the other against 5mM Tris pH 7.5 for 16h, at 4°C and subsequently incubated under assembly conditions, the sample dialyzed against 150 mM KCl yielded homogeneous fibrils, while that dialyzed against 5mM Tris in the absence of KCl yielded homogeneous ribbons. No seeding was used in any of the samples.

All spectra were recorded on uniformly labeled ^13^C, ^15^N human α-synuclein fibrils, with the exception of the PAIN spectrum which was obtained from a 1∶1 mixture of ^15^N labeled and ^13^C labeled monomers. The non-labelled positions were at natural isotopic abundance.

### Solid-state NMR

The solid-state NMR spectra were recorded on a Bruker AvanceII+ spectrometer using 3.2 mm triple resonance probes at a static magnetic field of 20.0 T. The spinning speed was set to 17 kHz and the sample temperature to 278 K. For spectra recorded under different conditions, the details are given with the figures. The carbon-carbon correlation spectra were recorded with a 20 ms DARR [43] mixing period and the nitrogen-carbon correlation spectra with 8 ms PAIN mixing. [33], [35] For the CANCO spectra selective adiabatic NCA-CP and NCO-CP steps were used. [44]–[48] 80 to 100 kHz SPINAL decoupling [49] was applied on protons in the direct and indirect dimensions. For the HSQC spectra refocused INEPT [50] steps were used and 5 kHz WALTZ decoupling [51] on nitrogen during acquisition. In the indirect dimension the heteronuclear J-coupling was decoupled using π-pulses.

Apodization with a shifted-sine bell function was applied in all dimensions and automated baseline correction in the direct dimension. All spectra were processed using topspin 2.1 (Bruker Biospin) and analyzed with the CcpnNMR software package. [52]–[54] Further experimental and processing details are given in Table S1 and Table S2.

## Discussion

We have structurally characterized two pure polymorphs of α-synuclein which were obtained under different salt concentration and otherwise identical conditions at neutral pH. Subtle changes in purification procedures, fibrillization buffers, orbital shaking and temperature can easily yield different fibril forms [5], [6], [55] or mixtures thereof. Sometimes, nominally identical preparation conditions lead to different polymorphs (compare spectral fingerprints in [25], [26] to [24]; as well as [22] to [6],[56]) while different conditions can still lead to a very similar polymorph (ribbons in [12]). NMR is a powerful method to assess the polymorphic homogeneity of a sample, even if other biophysical properties can also be very different between polymorphs.[12] The fact that the same polymorph can be reproducibly obtained, and that the samples are constituted by a single polymorph, as detected by NMR, laid the basis for a functional studies of the two forms.[12]

In addition to the analytical function, NMR is shown to lead to detailed structural information. Both forms, ribbons and fibrils, contain β-sheet elements, but their number, length and distribution differs greatly. The structural organization of the **ribbons** can be described as a succession of β-strands, interrupted mainly by glycines, with a larger turn being present in two locations, around amino acids 21 and 81. The middle part, between residues 44–57, reveals a loop connecting the two structures regions. In contrast, in the **fibril** form this part is fully structured; and the succession of β-strands, which is shorter than in the ribbons, runs from residue 38 to 94, without major interruption. Only around residue 74, three positive secondary chemical shifts are observed, indicative of a larger turn. Regarding the N-terminal portion, only residues 16–20 could be observed and form a β-strand. The fibrils have, in terms of overall organization, a pattern closer to other polymorphs described in the literature. In detail, however, the polymorphs are different, as the chemical shifts differ significantly (see comparison in [21]).

A structuration of the N-terminal region is unique to the **ribbon** form and has not been observed for any other polymorph described for α-synuclein. All other forms which secondary structure has been analyzed by NMR do display either static or dynamic disorder there, as revealed by the absence of the corresponding signals in the cross-polarization spectra.

**Figure 8:**
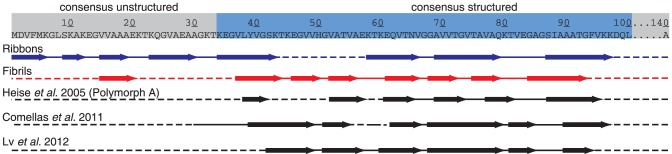
Secondary structure of the two polymorphs described here compared to two other polymorphs of human α-synuclein [6], [24] and one form of mouse α-synuclein. [**16**] The secondary structure for ribbons and fibrils is based on secondary chemical shifts and TALOS predictions. On top, the region that is usually assumed to form the fibrillar core is marked in blue. [31]

NMR structural data has been published for other polymorphs, and secondary structure organization has been proposed based on chemical shifts. Figure 8 shows the comparison of the secondary structure elements with the one of several published solid-state NMR assignments for different α-synuclein polymorphs. All human and mouse α-synucleins feature mainly β-sheet secondary structure, but the number and position of them differs greatly. The structural features of the fibril form resemble more the other polymorphs described in the literature. However, they display important chemical-shift differences to both forms for which sequential assignments are available. [7], [20], [24]

The observation of a short β-sheet in the N-terminal part is unique to the fibril form. All other forms which secondary structure has been analyzed by NMR do display either static or dynamic disorder at this position, as revealed by the absence of the corresponding signals in the spectra.[16]. Interestingly, the structurally conserved β-sheet-rich motif from residues 61–80, postulated by Lv *et al.* [16], is not conserved for the fibril form. In this region, we find strongly positive secondary chemical shifts for residues 73–75, indicating a pronounced arc or turn.

In the fibril sample, we observe peak doubling for a segment towards the C-terminal portion of the assigned region. Peak doubling points to structural heterogeneity and is a clear indication for a twofold conformation for the corresponding residue. Peak doubling has been observed for several proteins, often when more than one molecule was found in asymmetric unit. An extensive discussion about multiple peaks per site in amyloids is given in ref.[57] and multiplication by polymorphism was seen in [58]. Other examples include the Alzheimer β-peptide with two molecules in the asymmetric unit [59], Polyglutamine fibrils [60], [61] and the Ure2p C-terminal globular domain, where two sets of signals were observed for residues located close to crystal-contacts which were only made by one out of two molecules. [62] The origin for peak-doubling could not always be traced as precisely, and it can also attributed to local disorder. Peak doubling has also been observed for some residues in mouse α-synuclein,[16] where residues 84,85,86,89,93 and 94 are doubled, which coincides with the doubled stretch described here for the fibrils. The fact that the intensities of the two doubled components was found to be almost identical (Figure 4) strongly favors the interpretation with two molecules per asymmetric unit. The peak doubling observed here for the fibrils might be connected to the observation that the unit-cell dimensions, as determined by x-ray fiber diffraction, are twice as large for the fibrils than the ribbons. [12] This may hint at the building block of the fibril being an α-synuclein dimer.

So far, all α-synuclein polymorphs investigated structurally seem to show an in-register parallel stacking, although a detailed, residue specific, investigation, as shown in Figure 5, has not been available. The findings that both forms show in-register contacts over the entire sequence excludes an organization as observed for HET-s, where the chain stacks on itself to form two or more layers along the amyloid axis. [28], [29]. If the N-terminal β-strand forms part of the amyloid fibril core will have to be clarified by further studies.

Non-uniform dynamic features are revealed by the different intensity profiles of the assigned cross signals in the two polymorphs. Besides the parts of the sequence located in turns, there are also remarkable differences between the intensities of cross peaks arising from residues in β-sheets.

A flexible C-terminal domain from about residue 100 forwards has been described for all α-synuclein polymorphs so far. The chemical shifts in our HSQC spectra coincide well with those in solution and the signals from the C-terminal domain show very narrow lines in the HSQC spectra.

## Conclusions

We have, using solid-state NMR, structurally characterized two polymorph-pure forms of α-synuclein which have recently been shown to differ as well in other biophysical properties, as their x-ray diffraction pattern, and also in their biological toxicity and their seeding properties,[12] adding to the emerging picture that different polymorphs may well be at the hallmark of different strains and phenotypes in synucleinopathies. The results, from related work,[11] that different α-synuclein polymorphs can differentially promote different tau inclusions in neurons promotes this view as well. Our results show that the structural differences, which are at the origin of biochemical and toxicological differences between polymorphs and probably relate to different strains of α-synuclein, are very significant. While both are characterized by a series of β sheets with a length between 3 to maximum 6 residues (or possible 10 residues counting the lightly colored residues of Figure 2), the extent and distribution of sheets is largely different.

## Supporting Information

Figure S1 **Aliphatic region of 20 ms DARR.** The spectra were taken from the “early” sample which represents a mixture of U [^13^C,^15^N] labeled α-synuclein polymorphs. The spectrum was recorded at 14.1 T static magnetic field and 13 kHz MAS.
(EPS)

Figure S2 **Intensity ratio for selected doubled peaks using 20 ms DARR spectra.** (a) and (b) show older preparations where a mixture of asyn polymorphs was obtained, (c) a preparation of pure uniformly labeled asyn fibrils, (d) mixed 13C/15N-labeled asyn fibrils and (e) is the data set discussed in the main text. Chain A is shown in grey and chain B in black. The errors are estimated from the experimental noise. Missing bars indicate that the peaks were too weak to be quantitatively analyzed. All residues show a ratio close to 1∶1 except for Gly86 and Tyr92 where the two forms may feature slightly different mobility.
(EPS)

Figure S3 **PAIN spectrum of uniformly [^13^C,^15^N]-labeled α-synuclein fibrils (black) and of ^15^N/^13^C mixed labeled α-synuclein fibrils (red).** (a) overlay of both spectra, (b) trace through the 2D spectra at δ(^15^N) = 125.5 ppm. The spectra are scaled to the same intensity and contour levels in (a) are plotted at the same cutoff. Both spectra were recorded at 20.0 T static magnetic field and 17 kHz MAS. All parameters of the PAIN transfers (rf fields, length of recoupling period etc) were kept the same in both experiments. (c) Identical trace than in (b), but the trace of the [^13^C,^15^N]-labeled spectrum is scaled to the expected intensity of the natural abundance background peaks. The mixed labeled PAIN peaks are roughly 20 times more intense than it would be if only natural abundance background signal was observed. This comparison excludes that the observed PAIN peaks are from intramolecular transfer between the enriched ^15^N amide nitrogens and ^13^C nuclei at natural isotopic abundance.
(EPS)

Figure S4 **PAIN spectrum of uniformly [^13^C,^15^N]-labeled α-synuclein ribbons (black) and of ^15^N/^13^C mixed labeled α-synuclein ribbons (blue).** (a) overlay of both spectra, (b) trace through the 2D spectra at δ(^15^N) = 123.9 ppm. The spectra are scaled to the same intensity and contour levels in (a) are plotted at the same cutoff. Both spectra were recorded at 20.0 T static magnetic field and 17 kHz MAS. All parameters of the PAIN transfers (rf fields, length recoupling of recoupling period etc) were kept the same in both experiments. (c) Identical trace than in (b), but the trace of the [^13^C,^15^N]-labeled spectrum is scaled to the expected intensity of the natural abundance background peaks. The mixed labeled PAIN is roughly 20 times more intense than it would be if only natural abundance background signal was observed. See also caption of Figure S3.
(EPS)

Figure S5 **^1^H-^15^N HSQC spectra of fibrils (red) compared to ribbons (blue).** Resonances visible in the ribbons spectrum belong to the last 40 C-terminal resonances. Additional, weaker, signals are observed for fibrils, which probably come from α-synuclein monomers. Black crosses mark some assigned resonances using the liquid state shifts as a reference ^49^, that are also visible in the ordered parts of α-synuclein fibrils.
(EPS)

Table S1 **Experimental details.** (a) marks a dataset that already used in ref (21) of the main text.
(PDF)

Table S2 **Experimental details.** (a) marks a dataset that already used in ref (21) of the main text.
(PDF)
