# Supplementary material for: Unlike Twins: An NMR Comparison of Two α-Synuclein Polymorphs Featuring Different Toxicity
Source: PLoS One. 2014 Mar 5;9(3):e90659. doi: 10.1371/journal.pone.0090659 (PMC3944079; doi:10.1371/journal.pone.0090659)
Supplement: Table S1 — Experimental details. (a) marks a dataset that already used in ref (21) of the main text. (PDF) [file pone.0090659.s006.pdf]

Experiment	DARR 20 ms			PAIN UL		PAIN NC	
**Sample**	**mixture**	**ribbons**	**fibrils (a)**	**ribbons**	**fibrils**	**ribbons**	**fibrils**
Spectrometer	600 MHz	850 MHz	850 MHz	850	850	850	850
Probe	3.2mm TR	3.2mm TR	3.2mm LLC	3.2mm TR	3.2mm LLC	3.2mm TR	3.2mm LLC
MAS / kHz	13	17	17	17	17	17	17
Measurement Time	17 h	20 h	24 h	17 h	19 h	4 d	4.5 d
Number of Scans	48	16	32	32	16	176	176
Interscan delay / s	2.5	2.5	3	2.5	2.2	2.5	2.2
**Transfer 1**	**HC-CP**	**HC-CP**	**HC-CP**	**HN-CP**	**HN-CP**	**HN-CP**	**HN-CP**
Time / ms	1	0.6	0.5	0.7	0.8	0.7	0.8
Field / kHz	77(H)/88(C)	76(H)/63(C)	63(H)/47(C)	67(H)/50(N)	64(H)/49(N)	67(H)/50(N)	67(H)/48(N)
Shape	tangent(H) D=15kHz	tangent(H) D=30kHz	tangent(H) D=13kHz	tangent(H) D=27kHz	tangent(H) D=26kHz	tangent(H) D=27kHz	tangent(H) D=27kHz
Carrier / ppm	99	45	101	130	120	130	122
**Transfer 2**	**DARR**	**DARR**	**DARR**	**PAIN**	**PAIN**	**PAIN**	**PAIN**
Time / ms	20	20	20	6	8	6	8
Field / kHz	13(H)	17(H)	17(H)	72(H)/46(C)/46(N)	71(H)/45(C)/44(N)	72(H)/46(C)/46(N)	70(H)/45(C)/45(N)
Shape	-	-	-	-	-	-	-
Carrier / ppm	-	-	-	5.05(H)/44(C)/130(N)	4.96(H)/57(C)/120(N)	5.05(H)/44(C)/130(N)	4.96(H)/57(C)/122(N)
**Transfer 3**	-	-	-	-	-	-	-
Time / ms							
Field / kHz							
Shape							
Carrier / ppm							
**t1 Increments**	512	1792	896	768	1962	768	1024
Sweep width / kHz	40	75	45	25	74	50	74
carrier / ppm	99	45	101	130	120	130	122
acq. time / ms	6.4	11.9	9.9	15.4	13.2	7.8	6.9
td proc	1024	4096	1024	2048	4096	2048	2048
window function	qsine 4	qsine 2.7	qsine 2.6	qsine 2.6	qsine 2.5	qsine 2.6	qsine 2.5
**t2 Increments**	1280	2560	1536	3072	3072	3072	3072
Sweep width / kHz	50	85.2	50	100	100	100	100
carrier / ppm	99	100	101	100	100	100	100
acq. time / ms	12.9	15.1	15.4	15.4	15.4	15.4	15.4
td proc	4096	8192	2048	8192	8192	8192	8192
window function	qsine 4	qsine 2.7	qsine 2.6	qsine 2.6	qsine 2.5	qsine 2.6	qsine 2.5
**t3 Increments**	-	-	-	-	-	-	-
Sweep width / kHz							
carrier / ppm							
acq. time / ms							
td proc	-	-	-	-	-	-	-
window function							
**Decoupling**	SPINAL64	SPINAL64	SPINAL64	SPINAL64	SPINAL64	SPINAL64	SPINAL64
Field / kHz	80	100	100	90	85	90	85
