# Supplementary material for: Unlike Twins: An NMR Comparison of Two α-Synuclein Polymorphs Featuring Different Toxicity
Source: PLoS One. 2014 Mar 5;9(3):e90659. doi: 10.1371/journal.pone.0090659 (PMC3944079; doi:10.1371/journal.pone.0090659)
Supplement: Table S2 — Experimental details. (a) marks a dataset that already used in ref (21) of the main text. (PDF) [file pone.0090659.s007.pdf]

Experiment	NCA		CANCO		HSQC	
**Sample**	**ribbons**	**fibrils (a)**	**ribbons**	**fibrils (a)**	**ribbons**	**fibrils**
Spectrometer	850 MHz	850 MHz	850 MHz	850 MHz	850 MHz	850 MHz
Probe	3.2mm Bruker	3.2mm LLC	3.2mm TR	3.2mmr LLC	3.2mm TR	3.2mm TR
MAS / kHz	17	17	17	17	13	17
Measurement Time	34 h	8.5 h	2 d	3 d	12 h	3 h
Number of Scans	16	16	12	16	32	8
Interscan delay / s	2.5	2.5	2.2	2.8	1.33	1.33
**Transfer 1**	**HN-CP**	**HN-CP**	**HC-CP**	**HC-CP**	**HN-INEPT**	**HN-INEPT**
Time / ms	1.2	0.8	0.3	0.3	d4 = 2.5	d4 = 2.5
Field / kHz	53(H)/36(N)	53(H)/39(N)	80(H)/62(C)	68(H)/52(C)		
Shape	tangent(H) D=21kHz	tangent(H) D=21kHz	tangent(H) D=32kHz	tangent(H) D=14kHz		
Carrier / ppm	122	120	41	44		
**Transfer 2**	**NCA-CP**	**NCA-CP**	**NCA-CP**	**NCA-CP**	**HN-INEPT**	**HN-INEPT**
Time / ms	5	5	5	5.5	d4 = 2.5	d4 = 2.5
Field / kHz	100(H)/21(C)/7(N)	90(H)/20(C)/6(N)	90(H)/8(C)/8(N)	85(H)/10(C)/6.5(N)		
Shape	tangent D=9kHz	tangent(C) D=4kHz	tangent D=3kHz	tangent(C) D=2kHz		
Carrier / ppm	57	59	57	58		
**Transfer 3**	-	-	**NCO-CP**	**NCO-CP**	-	-
Time / ms			5	5.5		
Field / kHz			90(H)/8(C)/8(N)	100(H)/11(C)/6(N)		
Shape			tangent D=3kHz	tangent(C) D=2kHz		
Carrier / ppm			188	170		
**t1 Increments**	384	768	100	104	1024	1024
Sweep width / kHz	20	30	12	10	6	6
carrier / ppm	122	120	41	44	122	121.9
acq. time / ms	9.6	12.8	4.2	5.2	85	85
td proc	1024	1024	256	256	2048	2048
window function	qsine 2.6	qsine 2.6	qsine 2.4	qsine 2.4	qsine 2	qsine 2
**t2 Increments**	1280	1280	68	68	4096	4096
Sweep width / kHz	50	50	5.3	5.25	20	20
carrier / ppm	100	100	123	120	4.9	4.94
acq. time / ms	12.9	12.8	6.4	6.5	102	102
td proc	4096	2048	256	256	8192	8192
window function	qsine 2.6	qsine 2.6	qsine 2.4	qsine 2.4	qsine 2	qsine 2
**t3 Increments**	-	-	1280	1280	Water supression	Water supression
Sweep width / kHz			50	50	MISSISSIPPI	MISSISSIPPI
carrier / ppm			100	100	55 ms	55 ms
acq. time / ms			12.8	12.8	6 kHz	6 kHz
td proc	-	-	4096	4096	-	-
window function			qsine 2.6	qsine 2.6		
**Decoupling**	SPINAL64	SPINAL64	SPINAL64	SPINAL64	WALTZ16	WALTZ
Field / kHz	85	90	100	85	5	6
